# Understanding Team Learning Dynamics Over Time

**DOI:** 10.3389/fpsyg.2019.01417

**Published:** 2019-06-19

**Authors:** Christopher W. Wiese, C. Shawn Burke

**Affiliations:** ^1^Georgia Institute of Technology, Atlanta, GA, United States; ^2^Institute for Simulation and Training, University of Central Florida, Orlando, FL, United States

**Keywords:** team learning, temporal dynamics, team learning behaviors, time, review

## Abstract

Organizations depend on the learning capabilities of teams in order to be competitive in today’s information-laden business landscape. Hence, it is not surprising that there have been tremendous efforts made to understand team learning within the past two decades. These efforts, however, have produced a cluttered literature-base that overlooks a fundamental aspect of team learning: How do teams learn over time? In this paper, we first synthesize the literature to develop a shared vocabulary to understand team learning dynamics. We then leverage research investigating how teams operate within the context of time (e.g., team development, performance cycles, emergent state development) and combine it with the extant team learning literature in developing an unfolding model of team learning. This comprehensive model addresses a noticeable gap in the extant literature by illustrating how teams learn over time. Finally, we put forth three grand challenges for the future of team learning research.

## Team Learning Dynamics Over Time

*“Learning and innovation go hand in hand. The arrogance of success is to think that what you did yesterday will be sufficient for tomorrow”* – C. William Pollard in *The Soul of the Firm*

“*Team learning is vital because teams, not individuals, are the fundamental learning unit in modern organizations. This is where ‘the rubber meets the road’; unless teams can learn, the organization cannot learn.”* – Peter M. Senge in *The Fifth Discipline*.

Learning is key to remaining successful in today’s business landscape. The pressure to change and evolve at a moment’s notice is higher than ever – and this pressure often falls on the shoulders of teams. Teams are a collection of individuals who are interdependently working to achieve a shared goal (Salas et al., 1992) and organizations have come to rely on teams that can learn in order to be successful (Edmondson et al., 2007). When teams do not learn, it is likely that the organization will suffer. For example, teams that fail to learn will take longer to bring a new product to market (e.g., Sarin and McDermott, 2003). Hence, it has become crucial in both practice and academia to better understand team learning in order to enhance effectiveness throughout the organization.

The abundance of team learning research in recent years has revealedtwo main concerns with the state of the science, the resolution of which serve as the aims of the current manuscript. The first aim of these efforts is to explicate a shared understanding of team learning terminology. Through a review of the literature, we organize team learning constructs into two broad categories: team learning outcomes and team learning processes. We define two different types of team learning outcomes. Specifically, we use the term *team learning* to refer to a shift in a team’s collective knowledge state and the term *team learning curves* to represent changes in performance due to team learning over time. Additionally, we assert that *team learning behaviors* (i.e., behaviors that aid in the development of collective knowledge) can be further delineated into three different types of behaviors: *intrateam, interteam*, and *fundamental learning behaviors*. The conglomeration of different terminologies prevent meaningful discussion of the state of the science and a synthesis of unequivocal phraseology is necessary if we wish to move team learning science forward.

**FIGURE 1:**
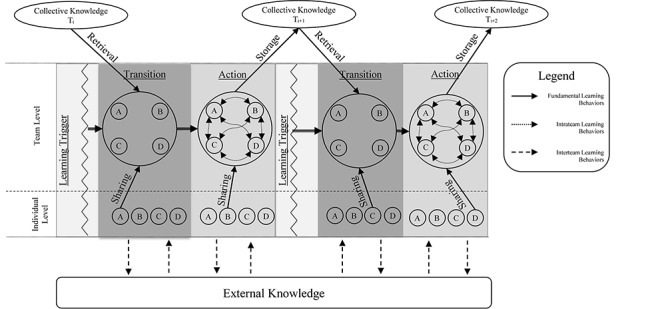
Unfolding model of team learning.

The second aim is to facilitate the understanding of how team learning occurs over time by presenting an unfolding model of team learning (Figure 1). By its very nature, team learning is a phenomenon that takes place over time and there is a critical need to understand teams in the context of time (e.g., Cronin et al., 2011). For team learning to occur, information needs to be shared amongst team members, discussed and scaffolded to existing knowledge, and stored in some way to be retrieved later. This process does not happen in a single moment, but in a series of interactions that unfold over time. While some of these aspects have been addressed in previous team learning models (e.g., Decuyper et al., 2010), our unfolding model of team learning provides a comprehensive framework of when, how, and what teams learn over time. By viewing team learning through our unfolding model (Figure 1), researchers and practitioners can reveal new insights on how learning develops over time and highlight factors that facilitate team learning and increase performance.

These two aims are accomplished as follows. First, we provide a detailed look into team learning terminology. The term *team learning* has been used to refer to various aspects of the team learning process – from behaviors that facilitate learning to shifts in collective knowledge to performance improvements over time. Hence, it is critical that, before presenting our unfolding model of team learning, a common understanding of language is achieved. Next, we describe how teams operate in the context of time. Here, we pull from the team development and team temporal dynamics literatures to discuss the evolution of teams in time and what that tells us about when, how, and what teams learn. Our efforts culminate in the presentation of an unfolding model of team learning, which leverages the existing literature to describe how teams learn over time. Finally, we set forth three grand challenges that need to be addressed in order to push the field forward. These challenges represent crucial gaps in the collective scientific knowledge state and, if addressed, will help teams learn and perform more effectively in practice.

## Team Learning Terminology

**FIGURE 2:**
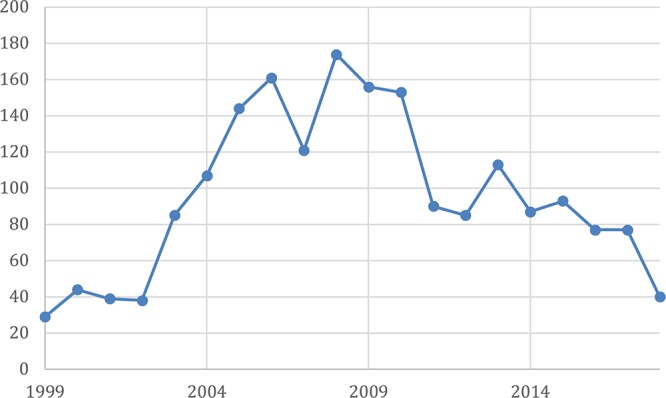
Number of articles using the keywords “team learning” or “group learning since 1999 in using Academic Search Complete, Applied Science and Business, Applied Science and Technology, Business Abstracts, Business Source Complete, PsycArticles, PsycINFO, and Vocational and Career Collection databases.

In order to understand how team learning occurs over time, it is first necessary to approach the topic with a shared vocabulary. While learning has been a key topic of research with respect to individual (e.g., Argyris, 1982) and organizational levels (e.g., Huber, 1991), a historical assessment of the literature suggests that team learning has only come into its own in the last two decades. Edmondson’s (1999) seminal article on team learning and psychological safety can be considered the catalyst of today’s team learning research landscape. In it, Edmondson defines team learning as a behavioral process – representing the cyclical process of seeking out (e.g., seeking feedback), gathering (e.g., asking questions), and discussing and integrating information (e.g., discussing errors). As shown in Figure 2, research in the organizational sciences focusing on team learning took off after the publication of Edmondson’s article (although reducing in recent years). An unintended consequence of this research thrust was the differential use of the term *team learning*. Like Edmondson, many use *team learning* to refer to behavioral processes (e.g., Gibson and Vermeulen, 2003; Wong, 2004), while others conceptualize it as changes in performance (e.g., Darr et al., 1995; Pisano et al., 2001), or shifts in collective knowledge (e.g., Ellis et al., 2003). Hence, before understanding how team learning unfolds over time, it is imperative that we approach this literature with a shared vocabulary.

Through a review of the literature, we collected different uses of the term “team learning” and summarize these findings in Table 1. Generally, there are two different thoughts on how to conceptualize team learning – as an outcome or as a process. Like previous authors, we adopt this distinction as an overarching categorization mechanism to better understand team learning. Elaborated upon in the next section, team learning as an outcome reflects the end result of learning processes, which fall into two distinct conceptualizations. Specifically, team learning outcomes could refer to either (1) changes in collective knowledge (i.e., *team learning*) or (2) shifts in performance (i.e., *learning curves*). Similarly, we found that team learning processes (i.e., *team learning behaviors*) can be further delineated into (1) *intrateam*, (2) *interteam*, and (3) *fundamental learning behaviors*. In the following section, we elucidate on this breakdown. It is our hope that our synthesis of team learning terminology will provide some much-needed conceptual clarity to the literature as well as facilitate understanding of our unfolding team learning model.

**TABLE 1:** Team learning terminology.

	**Term**	**Definition**	**Citations**
Outcomes	Team learning	Shift in the team’s collective knowledge state	Ellis et al., 2003; Wilson et al.,2007
	Learning curves	Shifts in team’s performance over time	Adler, 1990; Darr et al., 1995; Pisano et al., 2001; Edmondson et al., 2003; Reagans et al.,2005
Processes	Fundamental learning behaviors	Basic learning processes that promote learning in teams.	Wilson et al., 2007; sharing, storage, retrieval
	Interteam learning behaviors	Behaviors that seek and integrate information from entities outside of the immediate team that promote team learning.	Wong, 2004; Bresman, 2010; boundary spanning, scanning
	Intrateam learning behaviors	Internal behavioral processes that teams engage in that build shared meaning from existing information, identify and fill in gaps in the team’s collective knowledge, as well as challenge, test, and explore assumptions.	Edmondson, 1999; Drach-Zahavy and Somech, 2001; Van den Bossche et al., 2006; Savelsbergh et al., 2009; asking questions, seeking feedback, exploration, experimentation

### Team Learning as an Outcome

In order for us to understand how teams learn over time, it is crucial to recognize that learning – across all levels of consideration – is a temporally infused phenomenon. It infers a shift in knowledge state – a knowledge trajectory from one point in time to another. It is only logical, then, that conceptualizations of team learning outcomes hold these similar temporal properties. That is, team learning outcomes need to reflect a change in collective knowledge over time. Our review of the literature suggests that this is typically approached in two ways. The first is what we call *team learning*, which is a shift in collective knowledge. It represents the purest form of learning, harkening back to philosophical discussions on individual knowledge gain (e.g., Cornford, 1935). Collective knowledge refers to information held by the team about the team and its surrounding system. As it is a characteristic of the team, collective knowledge does not reflect knowledge held by any particular team member, but knowledge held by the team as its own united entity. For example, collective knowledge should remain intact when a member leaves the team. As Wilson et al. (2007) wrote “If an individual leaves the group and the group cannot access his or her learning, the group has failed to learn” (p. 1042–1043). Hence, in order for shifts in collective knowledge to occur, it is necessary for team members to interact and integrate individually held information into the team’s collective knowledge state.

While our conceptualization of team learning represents the most direct form of learning in teams (Kozlowski and Bell, 2008), it is nearly impossible to assess directly. One would need to identify the exact moment knowledge moved from an individually-held property to a team-held property. It is not surprising, then, that proxies such as team shared mental models and transactive memory systems are more commonly used to infer team learning. Both team mental models and transactive memory systems reflect the current state of the team’s collective knowledge, albeit in different ways. Team mental models represent the collective understanding of various aspects of the team’s operational system with respect to both content (what the teams know) and structure (relationship between different knowledge elements). Team learning can be inferred from team mental models in two ways, either mental model similarity at a single point in time or tracking mental model convergence over time (e.g., McComb, 2007; Santos and Passos, 2013).

Another proxy of team learning are evaluations of transactive memory systems. Transactive memory systems are representative of a shared information encoding, storing, and retrieval process among team members (Wegner et al., 1985; Wegner, 1986) and reflect who knows what on a team (Ren and Argote, 2011). Teams develop an understanding of their knowledge network through the cross-pollination of knowledge. In other words, team learning is a necessary prerequisite of transactive memory system development (Ellis et al., 2008) and, as such, transactive memory systems can be used as an indirect indicator of team learning.

It is also important to note that team learning has been inferred through changes in performance/effectiveness metrics, or *learning curves*. Much like team mental models and transactive memory systems, learning curves represent a consequence of team learning. The most pragmatic sign that teams are learning is increased performance due to the application of collective knowledge. Most often, this research has focused on efficiency indices. For example, decreases in task completion times (e.g., Pisano et al., 2001; Edmondson et al., 2003; Reagans et al., 2005) or decreased costs (e.g., Adler, 1990; Darr et al., 1995) are common metrics when studying learning curves. Still, whether it is speed, cost, or effectiveness, improvements in performance metrics are indicative that the team has learned. They have incorporated information into their collective knowledge and have subsequently applied that knowledge to improve the speed or performance of their collective action.

### Team Learning as a Process

Team learning over time (i.e., shifts in collective knowledge) are process-driven, which is how much of the literature has conceptualized team learning. Indeed, this is exactly how Edmondson (1999) characterized team learning – as an ongoing behavioral process. Over the years, the research on team learning has evolved, looking at various types of team learning behaviors. Table 2 provides an overview of what was found with respect to the different actions that have fallen under the label of team learning behaviors. Here, the term *team learning behaviors* is used to encapsulate all of the actions that aid in the development of collective knowledge. These actions, however, are not qualitatively similar. To better represent the nuances of team learning behaviors, we break them down into three different types: intrateam, interteam, and fundamental learning behaviors. In the following, the rationale behind this breakdown is briefly described by comparing and contrasting the three types of team learning behaviors.

**TABLE 2:** Overview of team learning behaviors with likelihood of occurring during the course of a team learning episode.

		***Learning episode***			
		
**Learning behavior**	**Definitions**	**Trigger**	**Transition**	**Action**	**Completion**
*Fundamental learning behaviors*					
Sharing	Actions team take to distribute information to team members	X	X	X	
Reception	Actively or passively listening to or receiving information	X	X	X	
Retrieval	Behaviors that clean collective knowledge from knowledge repositories (Wilson et al., 2007)		X		
Storage	Behaviors that support the maintenance and retention of collective knowledge over time (Wilson et al., 2007)				X
*Interteam learning behaviors*					
Scanning	Surveying the external environment for information relevant to the team’s task (Wong, 2004; Bresman, 2010)		X	X	
Boundary spanning	Obtaining information from individuals outside of the team (Ancona and Caldwell, 1992)		X	X	
*Intrateam learning behaviors*					
Asking questions	Seeking new or clarifying information (Edmondson, 1999)		X	X	
Seeking feedback	Seeking feedback from members internal to the team (Schippers et al., 2003)	X	X	X	
Exploration	Seeking out new knowledge and information (March, 1991)			X	
Experimenting	Collectively testing ideas and assumptions that deviate from pre-existing standards (van Woerkom, 2003)			X	
Discussing errors	Sharing and discussing errors the team has made (Van Dyck, 2000)		X		
Co-construction	Collaborative conversations that refine, build, or otherwise modify collective knowledge by producing new meaning (Savelsbergh et al., 2009)			X	
Constructive conflict	Discussion designed to resolve divergence in interpretations and opinions.			X	
Reflexivity	Reviewing and reflecting on previous team functioning (Schippers et al., 2013)	X	X		

First, *intrateam learning behaviors* are illustrative of the internal processes teams engage in that build shared meaning from existing information, identify and fill in gaps in the team’s collective knowledge, as well as challenge, test, and explore assumptions. This is representative of how most of the literature has operationalized team learning processes (Bresman, 2010). Examples of intrateam learning behaviors are: asking questions, experimenting, discussing errors and outcomes, constructive criticism, and exploration (e.g., Edmondson, 1999; Drach-Zahavy and Somech, 2001; Savelsbergh et al., 2009). Intrateam learning behaviors do not necessarily reflect the actions of sharing information with the team, but, instead, how the team obtains new information from their fellow team members and how that information is integrated into their collective knowledge. In other words, they are the knowledge obtaining and scaffolding processes that occur within the immediate team. However, information and insight may not only be provided by those from within the team, but outside the team as well.

*Interteam learning behaviors* occur when teams seek and integrate information from individuals outside the immediate team. While some of these behaviors (e.g., asking questions, seeking feedback) may be indistinguishable from intrateam learning behaviors, the consequence of these actions is absolutely different. Individuals outside the team are likely to bring new and different perspectives to the team’s dynamic compared to internal team members (Wong, 2004). On the one hand, these sorts of behaviors can be helpful. Fresh eyes can promote innovation and help teams better understand complex problems. New perspectives can be gleaned from individuals who are unfamiliar with the team’s current situation or individuals who can provide expert feedback (e.g., Ancona and Caldwell, 1992; Perry-Smith and Shalley, 2003; Hülsheger et al., 2009). On the other hand, integrating novel and unique information may produce drastic shifts to the team’s collective knowledge. While these drastic shifts may eventually be helpful, teams may initially experience decrements in coordination and increases in conflict (e.g., Jehn, 1995). Further, interteam learning behaviors not only differ with respect to who is providing information, but they also encapsulate learning processes not covered by intrateam learning behaviors. For example, in order to know what external knowledge is out there and subsequently act upon it, teams must engage in boundary spanning behaviors, which does not have a clear parallel in intrateam learning behaviors. Because of these differences with respect to both action and consequence, we distinguish between intrateam and interteam learning behaviors.

Lastly, *fundamental learning behaviors* represent the basic learning processes that promote learning in teams (Wilson et al., 2007). Unlike intrateam and interteam learning behaviors, fundamental learning behaviors are actions that individual team members take to share, store, and retrieve information. Our conceptualization of fundamental learning behaviors is an adaptation of the work by Wilson and colleagues. While we conceptualize both storage (i.e., behaviors that maintain collective knowledge over time) and retrieval (i.e., behaviors that glean knowledge from repositories) similarly, we diverge from Wilson et al., in our conceptualization of sharing. Wilson et al., conceptualizes sharing as behavioral processes that encompass most actions regarding the dissemination and integration of information within a team. We simply hold that sharing represents the actions teams take to make their fellow members aware of individually held information. Fundamental behaviors are distinct from intrateam and interteam learning behaviors as they exclusively represent how knowledge is transported across time. While, sharing represents how knowledge is transported from the individual to the team, storage behaviors are illustrative of how collective knowledge is preserved across time. Similarly, retrieval processes are those which represent how collective knowledge is transferred from repositories to the team’s awareness.

In the preceding section, the literature was synthesized to develop a shared understanding of team learning terminology. In addition to creating a shared language for those researching team learning, team learning terminology plays an important role in discussing the integrative, dynamic model of team learning presented herein (see Figure 1). With an established terminology, how teams operate in the context of time is presented next. Specifically, in the following section, team development and temporal dynamic theories are discussed in light of how these perspectives can inform the what, when, and how of team learning.

## Teams (And Team Learning) in Time

Teams are, in a word, dynamic. They develop; they change; they evolve. Researchers have been discussing teams in the context of time for at least three decades (McGrath, 1986; Cronin et al., 2011) and there has been much theoretical progress, which can be leveraged to better understand team learning dynamics. Specifically, work advancing our understanding of how teams develop (e.g., Team Development Theories, Tuckman and Jensen, 1977; Gersick, 1991), when teams engage in certain behaviors (e.g., Marks et al., 2001), and the nature of emergence (e.g., Kozlowski, 2015) can directly inform the dynamic nature of team learning. In the following section, we describe how team development theories shed light on *what* teams are learning, how temporal team process phases describe *how* teams are learning, and how understanding the nature of emergence can highlight *when* teams are learning.

### Team Development Theories and *What* Teams Learn

Some of the earliest work on understanding how teams operate over time comes from team development theories. These theories seek to understand the processes teams go through from their initial conception to their eventual disbandment. Most development theories can be classified as being grounded in either a linear growth model or a punctuated equilibrium model (Garfield and Dennis, 2012). Linear growth models describe team development as a series of ordered distinct phases, where teams accomplish particular goals within each stage. For instance, Tuckman’s (1965) model describes four stages where teams get to know each other (forming), begin to form a common understanding of the task landscape (storming), develop norms for task accomplishment (norming), and finally engage in task work (performing). In contrast, punctuated equilibrium models focus less on the order in which activities occurs and more on the timing of intense action. Typified by Gersick’s (1991) punctuated equilibrium model, team development is conceptualized as a period of activity at the team’s onset, followed by a period of inertia until the team reaches the midpoint of their performance cycle. At this point, teams reflect on their performance and reconsider their current strategies, culminating in a frenzy of team activity. This is followed by another period of inertia, with the team remaining relatively stable until the team disbands.

To varying degrees, team development theories speak to *what* teams are learning during specific stages in their development. Presently, we use Kozlowski et al. (1999) process model of team compilation as an illustrative case. This model suggests that team members learn different content at each of the four proposed phases. In the first phase, teams develop foundational knowledge that will facilitate knowledge growth in future stages. They form interpersonal communication networks, develop a shared understanding of the team’s task and requisite requirements (e.g., goals, task expectations), and a general sense of the team’s climate. In the second phase, teams begin to learn about team performance dynamics and member task-competencies. Specifically, team members begin to engage in task work, which conveys to other team members how performance will be completed and illuminates the capabilities of their teammates. As teams transition to the third phase, team members learn how their respective roles are interconnected. In other words, they learn about the coordination requirements of the task; who they will have to coordinate with, what they will need to coordinate about, and when/how this coordination will take place. Teams really come into their own during the fourth phase. Here, teams develop an understanding of multiple task-networks describing *who* and *how* to interact with about *what* under varying external contingencies. It is important to note that, while Kozlowski et al. (1999) proposed that initial stages of development are individually-focused and become more collective-focused over time, we propose the content (e.g., interpersonal knowledge, task competency) of what is being discussed becomes a part of the team’s collective knowledge repository. For instance, if Stan asks Lee a question that helps develop an understanding of communication styles, it is likely that the conversation is also observed by Gail and Simone – becoming part of the team’s collective knowledge.

While we do support the idea that teams typically learn basic knowledge before more advanced knowledge, the Kozlowski et al., model was an illustrative case and does not represent the definitive order of what teams learn. Instead, readers should take away that teams learn different content over time, which is often influenced by where they are in their developmental process. Next, we discuss what the temporal dynamics research on teams can tell us about *how* teams learn.

### Temporal Team Process Phases and *How* Teams Learn

While team development theories attempt to explain key considerations across the team’s lifecycle, time can also be used to help explain how teams accomplish their goals on a much smaller temporal scale. In their seminal paper, Marks et al. (2001) set out to describe the behaviors teams engage in during different periods of time (called performance episodes) as they seek to attain their goals. The framework that Marks et al. (2001), set forth has become the standard way to understand team processes over time and can be leveraged to address *how* teams learn over time.

Two fundamental contributions of the Marks et al. (2001) paper are utilized presently to help understand *how* teams learn in time. First, the authors apply a temporal layer to the concept of team performance episodes. Popularized in the 1990s, team performance episodes are discernable blocks of time where teams engage in goal directed activity (Mathieu and Button, 1992). These performance episodes are not independently occurring, nor are they similar in structure. In other words, a team can be engaged in multiple performance episodes related to different tasks simultaneously and the time taken to complete each can vary (e.g., McGrath, 1991). Marks et al. (2001) suggested that a temporally-based classification system can be derived from types of activities teams are engaging in that facilitate goal accomplishment. Specifically, they suggest two phases of team processes: action and transition phases. A sub-episode, a period of time within a particular performance episode, is classified as an action phase if the team is directly working toward accomplishing their goal (i.e., engaging in taskwork). In contrast, transition phases are when the team takes a respite from taskwork – taking time to reflect on their past performance and plan for the future.

Second, Marks et al. (2001) supplement this distinction by positing that there are certain types of behaviors that teams typically engage in within and across these phases. That is, these temporal phases can be used to describe *how* teams go about accomplishing their goal. Specifically, there are processes that generally occur during transition phase (transition processes), action phase (action processes), and across these phases (interpersonal processes). In short, teams are more likely to engage in behaviors that support the reflection and evaluation of goal progress during the transition phase (e.g., mission analysis, goal specification), behaviors that directly support goal accomplishment during the action phase (e.g., coordination, monitoring progress toward goals), and behaviors that facilitate team and task-work across these phases (e.g., conflict management, affect management). In essence, the Marks et al. (2001), framework provides a temporal structure in explaining how teams accomplish their goals.

This framework is leveraged to better understand *how* teams learn across time. First, the idea of performance episode phases is directly applicable to how team learning occurs. Specifically, *team learning episodes* can be thought of discernable periods of time where teams become aware of and integrate information into their collective knowledge state. Much like Marks et al.’s (2001) model, these episodes can be characterized by transition and action phases, where transition phases are those where information makes its way to the team’s collective awareness and the action phase represents the time where teams discuss and debate that information to the point in which it becomes part of their collective knowledge state. Additionally, as discussed earlier, there are many different types of learning behaviors – some of which are more likely to occur within a specific phase and others which are likely to occur across all phases. Table 2 provides an overview of where we believe these learning behaviors may be most likely to occur. Our rationale for this is elaborated upon later.

### Multilevel Emergence and *When* Teams Learn

In this section, the process of emergence is described and how emergence relates to when teams learn is discussed. Generally, emergence is used to describe the bottom-up process, wherein lower level characteristics manifest to higher order phenomenon through interactions (Holland, 1998; Morgeson and Hofmann, 1999; Kozlowski and Klein, 2000). As such, it is a multilevel phenomenon that is process oriented and takes place over time (Kozlowski et al., 2013). Within teams, it is the interactions between team members that drives the development of team-level emergent states such as psychological safety, trust, and cohesion. The speed at which emergence occurs depends on several factors. For instance, there are conceptual differences between different types of emergent states that may influence how quickly they emerge (Kozlowski et al., 2013). Another factor that drives emergence is exposure to particular events (or triggers). For instance, teams need to engage in some risk-taking behaviors to judge their fellow teammates reactions and develop psychological safety (Edmondson, 2004). Further, Kozlowski et al. (2013) discuss how triggers could lead to swings in cohesion over time. Hence, the speed and pattern of emergence varies based on the conceptual underpinnings of the construct in question as well as the exposure to triggers – both of which are factors to consider when thinking about the manner in which team learning emerges.

**FIGURE 3:**
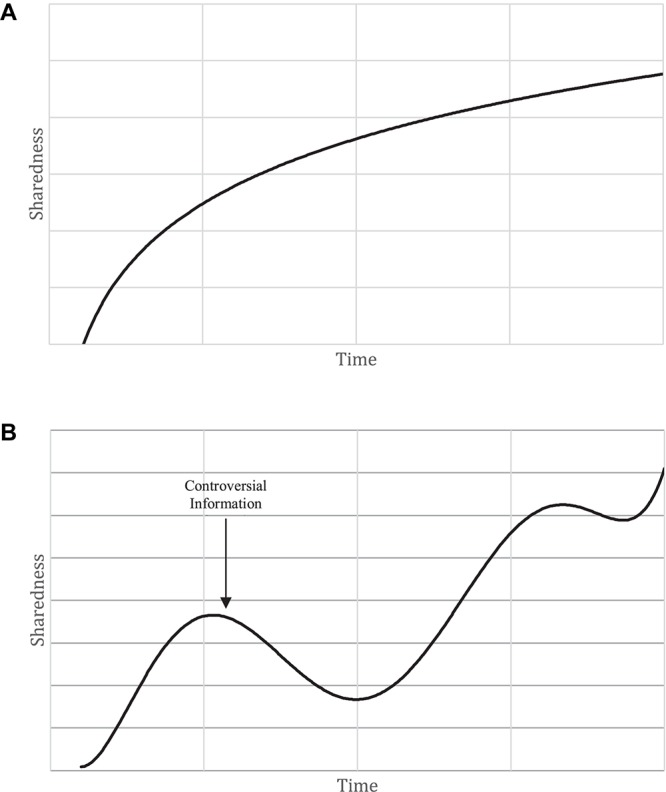
**(A)** Illustration of monotonically increasing sharedness of interpersonal interaction pattern over time. **(B)** Illustration of how controversial information impacts sharedness of collective task knowledge over time.

Team learning is an emergent state. It stems from team processes (process-driven) that integrate individual information into the team’s collective knowledge state (multi-level), which occurs over a period of time (over time). The speed in which team learning emerges is highly contingent on what is being learned. Going back to the Kozlowski et al. (1999) framework, teams will quickly learn about interpersonal interaction patterns, whereas learning about team member task competencies may take more time. Interestingly, the content of what is being learned will also influence the pattern of emergent states across time. For example, learning about interpersonal interaction preferences should create a monotonically increasing pattern when using a shared knowledge index of team learning (Figure 3A). As long as membership does not change, knowledge about the social interaction patterns of the team should remain stable over time. Conversely, a similarity index used to capture the team’s agreement on how a new, controversial piece of information influences the task landscape my result in a more dynamic pattern (Figure 3B). While agreement was previously a characteristic of the team’s past collective knowledge state, differences in opinion could drive team members apart and it will take time to come to a shared understanding again. These examples also suggest that events may be the catalyst of team learning; we call these events *learning triggers*. We define learning triggers as events in which the team inspects or questions their collective knowledge state in some way. It could be due to new information coming to light, a change in task demands, or an external entity bringing new information to the team.

## Unfolding Model of Team Learning

In this section, we explain our unfolding model of team learning (Figure 1) in detail. This model was developed by leveraging the extant literature on team learning and integrating it with our current understanding of teams in time. In the following, we discuss *when* teams learn by elaborating upon the catalyst of team learning, team learning triggers. Following this, we describe *how* teams learn by first describing what happens within the two phases of team learning: transition and action. Next, we elaborate on *how* teams deal with and integrate information by placing team learning behaviors in the context of time. Finally, we extend our model outside of a single learning episode and discuss what learning looks like over longer periods of time.

### Learning Triggers

As mentioned earlier, team learning triggers are events which cause the team to inspect their current collective knowledge state. These events have the potential to generate change in the team’s collective knowledge state (i.e., generate team learning). As such, it is important to discuss where these triggers come from and how they set teams on a path of learning. Team learning triggers come from a variety of sources, the likelihood of which partially depends on where the teams are in their development. During initial phases of the team’s development, team learning triggers are likely to come from individual sources. For example, imagine a new product development team that has never worked together. Individual team members will need to share their communication preference with their teammates in order to develop collective knowledge with respect to the team’s interpersonal network. Team leaders can also provide a source of learning during the initial phases of development. Using the same example, a product team’s leader will provide goals and expectations for team such as providing clear deadlines and relationship expectations between team members.

As teams develop, learning triggers may begin to come from team level sources. For example, some team developmental models suggest that teams reflect on their performance progress and establish new goals for future performance after a period of time together (e.g., Gersick, 1991), which can be used to stimulate learning. Further, the learning process may be triggered by process-oriented events such as making mistakes or facing difficult challenges, which typically occur after initial stages of development. For example, product development teams may face challenges that cannot be addressed by their team’s current collective knowledge state (e.g., Edmondson and Nembhard, 2009). In these cases, teams may seek the opinion of external sources of knowledge (e.g., Marrone et al., 2007), which can stimulate the process of learning.

Lastly, some team learning triggers are unpredictable in nature and could occur at any time during the team’s life-cycle. Unexpected changes are a common characteristic of many teams (e.g., SWAT teams, Bechky and Okhuysen, 2011; military teams, Burke et al., 2006) and teams need to adapt and learn in order to respond to these changes (e.g., London and Sessa, 2007; Oertel and Antoni, 2014). No matter where the trigger is coming from, the presence of a trigger does not necessarily mean that the teams will learn. Teams exposed to new information can easily dismiss it or may not be aware that a trigger has occurred. In order for teams to learn, team members must become collectively aware of new information and then integrate it into their collective knowledge. This process is described in detail in the next two subsections.

### Transition/Action Phases

Team learning triggers can generate *team learning episodes*, which are discernable periods of time where teams becomes aware of and integrate information into their collective knowledge state. As alluded to earlier, there are two temporal phases that occur within these episodes. During the transition phase, the information embedded in the team learning trigger must reach a state of collective awareness within the team. Having the team be aware of this new information is a crucial prerequisite for team learning to occur. As Wilson et al. (2007) put forth, if knowledge is lost when a team member leaves the group, the team has not learned. Hence, it is imperative that all team members are aware of the new information at the onset of the learning process. During this phase, the team’s current collective knowledge state also needs to be brought into the team’s collective awareness. As mentioned earlier, team learning triggers are events that call into question the team’s collective knowledge state. Once collective awareness is achieved, teams move onto the action phase, where they begin the process of integrating new knowledge into their collective knowledge state. As elaborated upon in the next section, teams scaffold the new knowledge onto their collective knowledge state through discussion, experimentation, conflict, and construction. These processes build new meaning and facilitate shifts in the team’s collective knowledge.

A quick illustrative case can highlight how this process unfolds. After creating a prototype of a new foldable smartphone, the marketing-lead on a new product development team receives consumer feedback that the malleable screen material is breaking down after repeated uses. In order for team learning to occur, the marketing-lead must not only provide this new information to the team at-large, but also remind the team how it relates to the previous conversations on what materials to use for their new smartphone (transition phase). This new information is then integrated into the team’s collective knowledge state (e.g., we cannot use this material on our new smartphones) and previously discussed alternative materials will need to be deliberated until a new decision is reached (action phase).

Thus far, the temporal structure of our unfolding model of team learning has been discussed. First, a team learning trigger occurs that contains new information that the team will consider. Next, this information makes its way to the team’s collective awareness (transition phase), which then leads to scaffolding information with respect to the team’s collective knowledge state (action phase). Team learning occurs once this information is integrated into the team’s collective knowledge state. However, team learning is an emergent state which is inherently process-driven and we would be remiss if the processes that facilitate team learning over time were not discussed. Specifically, we next discuss *how* team learning occurs and, in doing so, highlight the temporal patterns associated with different learning behaviors.

### Learning Behaviors Over Time

Earlier, we classified team learning behaviors into three categories (intrateam, interteam, fundamental) which facilitate team learning in different ways. While this classification helps clarify different types of learning behaviors, it does not necessarily speak to when these learning behaviors are likely to occur. Hence, in this section, we walk through a team learning episode to not only highlight how these behaviors facilitate team learning, but also when they are likely to do so. Specifically, we discuss when team learning behaviors are likely to occur as part of a learning trigger, during the transition and action phase, and the eventual emergence of team learning. We summarize the likelihood of these learning behaviors occurring in Table 2.

#### The Learning Trigger

First, team learning behaviors can serve as the catalyst of a team learning episode. As highlighted earlier, simply sharing (fundamental learning behavior) information about communication preferences can trigger a learning episode that can lead to team learning. A learning episode may also be triggered by teams reflecting on past performance or discussing errors that have occurred. Research has shown that the act of reflection can stimulate learning, especially when teams are not performing well (e.g., Schippers et al., 2013). Reflection often brings to light errors that teams have made in the past, but have not had the opportunity to discuss. It is important to note that these reflective behaviors may only *trigger* a learning episode. That is, shining a light on errors or performance may not necessarily lead to adaptation and adjustments (e.g., Kluger and DeNisi, 1996). In order to learn, teams need to engage in behaviors typified in the transition and action phases.

#### Transition Phase

As teams move onto the transition phase, teams need to recall their collective knowledge state as well as ensure collective awareness, which require different learning behaviors. First, teams engage in retrieval behaviors to bring collective knowledge into the team’s collective awareness. Retrieval is a fundamental learning behavior where team members search for, gather, and recall previously learned knowledge (Wilson et al., 2007). Second, teams must not only retrieve their collective knowledge state, but also guarantee that the team is aware of this new information. This is done through fundamental learning behaviors (sharing, receiving) as well as interteam (e.g., scanning) and intrateam (e.g., asking questions) learning behaviors. Awareness of the new information is spread throughout the team by simultaneous engagement in sharing and receiving behaviors. Sharing promotes collective awareness by directly telling fellow team members of new developments. Conversely, reception is passive in nature, involving the listening to and the receiving of new information. If there are questions concerning the accuracy or legitimacy of this new information, team members may seek more information through internal (e.g., asking questions) or external (e.g., scanning, boundary spanning) sources.

#### Action Phase

As the team enters the action phase, they begin to engage in behaviors that facilitate the integration of new knowledge into their collective knowledge state. One way teams can do this is by engaging in constructive conflict behaviors. Constructive conflict behaviors are those that bring about team members’ opinions on new information and discussion of these likely differing opinions. It helps resolve disagreements and gets them on the same page before going forward. Relatedly, teams can engage in experimenting behaviors to test out hypotheses on the way to resolving conflicting opinions (e.g., Gibson and Vermeulen, 2003). Co-construction is another prime example of a learning behavior that occurs during the action phase. Co-construction occurs when team members collaboratively work together to bring new meaning to pre-existing ideas (Van den Bossche et al., 2006). In effect, team learning behaviors occurring within the action phase directly support the integration of new and existing knowledge.

#### Concluding a Learning Episode

Finally, once the team has integrated this new information, there is one last team learning behavior which is necessary before it can be said that the team has learned. Specifically, the team must engage in storage behaviors. These actions are where teams place collective knowledge into some form of a repository to be retrieved later (Wilson et al., 2007). Here, repositories are broadly defined. Team members can store knowledge in physical repositories (i.e., paper copies, digital databases) or, more often, knowledge is stored cognitively (i.e., in memory). These actions help sustain and retain the conclusion of the learning process. Indeed, if new information is lost, it is difficult to say that the team experienced any learning.

It is important to note that some team learning behaviors can support learning across different phases. For instance, sharing information is a crucial learning behavior in the transition phase as it helps teams become aware of new information as well as the action phase, where team members are expressing their opinions in constructing new knowledge. Also important is the idea that our framework does not propose that learning behaviors exclusively occur during a particular phase. Much like the Marks et al. (2001), framework, we suggest that team learning behaviors are *likely* to occur during these phases. In the next section, the presented model (see Figure 1) is expanded beyond learning episodes to discuss team learning over longer periods of time.

### Learning Over Time

Up to this point, team learning has been discussed as it occurs from a micro/learning episode perspective. That is, the discussion has focused on how a piece of information is integrated into and changes the team’s collective knowledge state. However, team learning takes place over the entire course of the team’s life cycle, which has implications for how learning fluctuates over longer periods of time. Namely, we address learning fluctuations with respect to learning episodes and learning patterns over time. First, the factors that influence the speed, length, and completion of learning episodes are delineated. Second, the manner in which different learning patterns may emerge over time is highlighted.

#### Team Learning Episodes

Not every team learning episode is the same. Some learning episodes will only last for a short while, whereas others may never be completed. The length of team learning episodes depends on a number of factors. First, learning episodes will last longer when the information is more complex. When teams face the challenge of integrating complex information into their collective knowledge state, they have a higher propensity to engage in information-processing failures (Schippers et al., 2014). Schippers et al. (2014) suggest three categories of information-processing failures where teams fail to (1) reveal/discuss information, (2) explain or scrutinize information, and (3) successfully integrate new information into their prior beliefs/current behaviors. Teams do make efforts to avoid these failures, however, doing so will prolong the team learning episode.

Second, team learning episodes may also be prolonged when teams are asked to integrate information that is conflicting or starkly divergent from the current state of their collective knowledge. Teams are comfortable maintaining the status quo and ideas that come into conflict with the status quo will be met with resistance (e.g., Janis, 1972; Whyte, 1989; Schulz-Hardt et al., 2002). Further, divergent information is more likely to engender differences in opinion, which will need to be resolved before the team can learn. Incorporating information that is not congruent with the team’s collective knowledge state may take more time, but there are also payoffs down the road. For instance, teams that are able to integrate this conflicting information may be more likely to perform better on creative or innovative tasks (e.g., Dahlin et al., 2005; Edmondson and Nembhard, 2009), which is ultimately worth the longer time it takes for them to learn.

Third, factors external to a particular team learning episode could prevent teams from completing that learning episode. As mentioned earlier, multiple learning episodes can occur simultaneously, overlapping each other and, potentially, conflicting with one another. Some team learning episodes may fall to the wayside as they are no longer prioritized in the grand scheme of the team’s agenda. For example, federally-funded research teams who are learning about different methods of securing funding in light of a looming governmental shutdown may cease learning about funding alternatives once a budget gets passed. Another external factor that could prevent completion of a team learning episode is change in membership. If a team member suddenly exits, it could slow down or halt the progress of learning.

#### Team Learning Patterns

By using similarity/dissimilarity indices of team learning (e.g., shared mental models), one can begin to observe patterns of learning over time. The forms the patterns take over time are influenced by a number of factors. First, much like team learning episodes, the complexity of the information being learned becomes a factor when considering the pattern of team learning over time. As mentioned above, the complexity of information could prolong a single learning episode. This idea can be extended to the similarity of team’s collective knowledge over time. Simple information could be integrated into the team’s collective knowledge state relatively quickly; producing a monotonically increasing pattern of similarity over time. Complex information, however, may (1) take longer to integrate into a shared mindset and (2) be more prone to disagreements along the way. These two scenarios will produce different learning patterns over time, with one illustrating a monotonically increasing pattern of similarity over time (albeit at a slower rate) and the other more variable pattern of similarity.

Second, the content of what is being learned may vary in its susceptibility to change. As mentioned earlier, teams can learn different content at different times during their development and throughout their performance cycle. Some of this content will remain stable across the course of their performance cycle whereas other content will be more likely to change. Similarity indices have been used to capture a variety of different types of knowledge (Cannon-Bowers et al., 1993) and can be used to capture the different patterns of similarity over time. For instance, similarity in knowledge about team characteristics may monotonically increase and remain consistent across the team’s lifespan (baring no membership change). However, this stability may not be mirrored when the content of collective knowledge concerns more tasked based information. For example, team member similarity with respect to budgetary allotments may change drastically over time as new information comes to light.

Inferred in many of these examples is the idea that learning triggers can influence the shape of learning patterns over time. Although not exclusively focused on learning, this idea can be extracted from Gersick’s (1991) punctuated equilibrium model. Within this model, teams experience a learning trigger in the form of time pressure that comes with the recognition that they are halfway through their performance cycle. This recognition spurs on team activity and, in a sense, learning. Unlike Gersick’s model, we proposed that teams experience multiple learning triggers throughout their performance cycle, which could lead to various patterns in learning over time. As indicated above, learning triggers may not immediately result in a shared understanding. Using a similarity index to model learning, learning triggers could result in either more similar mental models or dissimilar mental models. As we discuss later, this has measurement implications for those looking to track learning patterns over time.

### Summary of Model

In the preceding section, we detail *what*, *when*, and *how* teams learn over time. Specifically, a temporal framework of how teams integrate new information into the collective knowledge states as well as the behaviors that facilitate this process was presented. We also described the manner in which teams learn over longer periods of time. Herein, the factors that may influence the length of learning episodes as well as the influences that shape learning patterns over time were discussed. In the next section, avenues for future team learning research are delineated. Specifically, three challenges are laid out for researchers interested in understanding team learning in the future.

## Challenges for Research

Team learning is a crucial aspect of what makes organizations successful, but there is still much that is not understood. In the previous sections, research on team learning and teams in time was integrated to produce an unfolding model of team learning. In this process, areas that may best serve as the next frontier of research on team learning were highlighted, but not necessarily explicitly stated. In this section, we explicitly state these areas in the form of three challenges for research that we believe are the logical next steps for researchers to address. These challenges are not necessarily the lowest hanging fruit – in fact – quite the opposite. They represent the most fundamental gaps in knowledge and practice that we believe future researchers will need to accomplish to advance the field.

### Team Learning Measurement

As mentioned earlier, measuring the point in which new knowledge becomes part of the team’s collective knowledge state is practically impossible. In the future, methods may exist whereby one can infer this conceptualization of team learning through subtle social cues (e.g., body language when a statement concerning the new state of collective knowledge is articulated), but these ideas are of no help to current research. Instead, research should focus on creating better methods by which to measure team learning proxies and modeling the team learning process over time.

#### More Frequent Measurement of Team Learning Proxies

This manuscript has presented team learning as an emergent state that is process driven, multi-level, and unfolds over a period of time. Unfortunately, measurement proxies to capture team learning (e.g., team mental models, transactive memory system) are not typically measured in a way to capture this emergence. Early research capturing team learning proxies measured these constructs once or twice through the team’s performance period (e.g., Roe, 2008; Zhou and Wang, 2010). This does not allow researchers to infer how team learning over a period of time unfolds. Hence, to better understand the emergence of sharedness or the development of transactive memory, researchers need to measure these team learning proxies multiple times throughout performance cycles. This, however, represents the practical roots of the challenge.

Measures of mental models and transactive memory systems are relatively intensive and disruptive. For example, card sorting programs are often used to capture both the content and structure in team member mental models (e.g., DeChurch and Mesmer-Magnus, 2010). This requires team members to cease what they are currently doing, open up the card sorting program, and sort the cards by making associations between cards before they can engage in taskwork again. Further, it is difficult to capture these cognitive emergent states in an unobtrusive way. Despite several calls for these types of measures (e.g., Rosen et al., 2011; Kozlowski, 2015) there has not been much development of unobtrusive, inexpensive measures of cognitive emergent states. Hence, we call upon researchers to measure team learning proxies more often throughout a team’s performance cycle, which could mean the development of unobtrusive measures that capture the team’s collective cognitive state.

#### Smarter Measurement of Team Learning

As^1^ team learning is a process-driven emergent phenomenon, it will be important to consider *where* learning episodes take place when trying to assess team learning and team learning behaviors. In this manuscript, we have taken a stance similar to that of other organizational scientists: that organizational phenomenon needs to be modeled with time in mind (Cronin et al., 2011; Lehmann-Willenbrock and Allen, 2018). In this effort, we have presented our unfolding model of team learning without addressing the influence of *where* these interactions take place. More specifically, the processes that drive team learning take place at various points within the team’s lifespan, however, there may be certain times in which behaviors that facilitate learning are more likely to occur.

Teams researchers understand that *where* team interactions occur plays an important role of the development of emergent states and can lend insight on how teams learn. This is especially important in the age of virtuality. Team dynamics do not occur purely in a face-to-face environment (e.g., Connaughton and Shuffler, 2007; Shuffler et al., 2010) and the degree to which team processes encourage team learning likely depend on the virtuality involved in team interactions. Hence, it is crucial for teams researcher to not only consider how team learning unfolds over time, but also recognize the where learning takes place could include the speed and quality of emergence. This calls for a smarter measurement approach to understanding the phenomenon – capitalizing on contexts where team learning is most likely to take place.

#### More Complete Measurement of Team Learning Model Over Time

In the model of team learning presented herein, several drivers of team learning were delineated. Specifically, teams experience a learning trigger, which is then followed by a series of behavioral processes that facilitate team learning. To be best of our knowledge, very few studies have examined how either triggers or behaviors influence team learning (or proxies of team learning) over time. A notable example is the work by Oertel and Antoni (2015), who investigated how transactive memory systems developed over time, finding that the effectiveness of different types of learning behaviors (e.g., knowledge-based, communication-based) in the development of transactive memory systems depended on *when* these behaviors were enacted.

Despite being a focal aspect of how emergent states develop, there is a relative dearth of research investigating how events (i.e., learning triggers) influence the sequence of learning behaviors teams engage in, let alone the development of team learning. For us, this represents the largest and most crucial gap in knowledge on team learning. In order to facilitate change in the team’s collective knowledge state, it is crucial to understand the catalyst of that change. However, there are several fundamental questions concerning team learning triggers that are currently unanswered. Are different teams equally aware of the same learning trigger? How do differences in learning triggers (e.g., content, intensity) influence how teams respond to these triggers (e.g., behaviors they engage in, speed in which they learn)? What can be done to enhance the clarity of learning triggers to facilitate subsequent learning episodes? The answers to these questions are unclear. Hence, we posit that studies need to be designed such that a more complete picture of the learning process is captured through measuring learning triggers, team learning behaviors, and team learning proxies longitudinally.

### Team Learning Content

Related to the previous challenge, there is a need to understand how the content of what is being learned influences the multiple aspects of team learning. Earlier in the manuscript, it was argued that the content of what the team is learning will influence how quickly the teams develop a shared understanding of that knowledge. The content of what is being learned may not only influence the speed at which information is learned, but also the rate in which collective knowledge is lost. A popular colloquialism applies here: *Use it or lose it*. The limited about of research that seeks to capture how teams learn over time investigates how teams gain/develop shared mental representation of the construct space – not how knowledge is lost over time. To address this issue, we challenge researchers to investigate this with respect to both the content of knowledge and the storage of behaviors.

Looking into *what* the team has learned may be predictive of how quickly that information is lost. Specifically, researchers interested in modeling knowledge loss over time need to think about how the content of collective knowledge is related to its usage and, subsequently, design measurement occasions around how quickly they believe this knowledge is lost. For instance, knowledge concerning interpersonal communication networks may never depart the team’s collective knowledge state as it is constantly used and reinforced. Conversely, collective knowledge with respect to a specific communication medium (e.g., how to use Slack) may dissipate over time with a lack of use. Further, the rate in which collective knowledge is lost may be influenced by particular storage behaviors that facilitate the maintenance of collective knowledge. For example, teams relying solely on cognitive repositories (i.e., memory) to retain collective knowledge may lose this knowledge quicker than teams that who rely on physical knowledge repositories (e.g., file systems, meeting notes, etc.).

### Disruptive Learning Triggers

Not all team learning triggers will have the same impact on team learning. As mentioned earlier, some team learning triggers are relatively simple in nature, such as the ones that stem from knowledge shifts with respect to interpersonal communication networks. However, other team learning triggers can be more disruptive and, consequently, have a large impact on team learning and subsequent performance. Team member exit and entrance are two of the most common and disruptive learning triggers teams can experience, yet seldom investigated (Liu et al., 2011). With respect to team member turnover, teams will have to undergo an intensive relearning period (van der Vegt et al., 2010). At the point of member exit, it is likely that the team has developed a shared understanding of routines and interaction patterns (Katz, 1982), which need to be re-established once one of the crucial nodes in their network is no longer present. Teams will also need to engage in a similar relearning period in the event of newcomers. Routines and interaction patterns will need to be adjusted and re-established to incorporate the new member. Currently, little research exists documenting how disruptive learning triggers influence team learning behaviors or team learning, which is why we believe it represents a pressing challenge for future research.

## Conclusion

Teams are the cornerstone of most organizations today and, hence, it is crucial that researchers and practitioners alike take the time and effort to understand teams better. One of the most crucial functions teams perform for these organizations is learning. As Senge and Peter (1991) pointed out nearly three decades ago, teams are the central learning unit of the organization and, consequently, organizational success will very much be determined by how well teams learn. Starting with Edmondson’s (1999) article, the literature on team learning began to grow and expand – budding off in different directions until the team learning literature landscape was cluttered and confusing.

This manuscript is an attempt to integrate the disparate research streams that contribute to our understanding of the dynamic nature of team learning. Herein, the literatures on team development, temporal process phases, and multilevel emergence are leveraged to present a path forward for understanding what, how, and when teams learn. In doing so, we provide a cohesive terminology and describe the ways in which team learning has been conceptualized in the literature. We extended the literature base by clearly delineating the intra- and inter-team learning processes, as well as fundamental learning processes. Next, we describe the role of team learning triggers and their differential impact across the temporal phases within team performance episodes. This information was then incorporated into an integrated model that can serve as basis for understanding the nuances of team learning in time. Finally, following from the presented model are three grand challenges that we believe are next steps for research on team learning. It is our hope that the description of the dynamic nature of team learning, the factors that impact it, and the model presented herein will serve to guide future discussions and push the field toward more consideration of the temporal aspects of team learning.

## Author Contributions

CW and CB contributed to the writing and theoretical development of the manuscript.

## Conflict of Interest Statement

The authors declare that the research was conducted in the absence of any commercial or financial relationships that could be construed as a potential conflict of interest.

## Footnotes

1 We would like to thank one of our reviewers for this suggestion.
